# Self-organization of frozen light in near-zero-index media with cubic nonlinearity

**DOI:** 10.1038/srep20088

**Published:** 2016-02-05

**Authors:** A. Marini, F. J. García de Abajo

**Affiliations:** 1 ICFO-Institut de Ciencies Fotoniques, The Barcelona Institute of Science and Technology, 08860 Castelldefels (Barcelona), Spain; 2 ICREA-Institució Catalana de Recerca i Estudis Avançats, Barcelona, Spain

## Abstract

Optical beams are generally unbound in bulk media, and propagate with a velocity approximately amounting to the speed of light in free-space. Guidance and full spatial confinement of light are usually achieved by means of waveguides, mirrors, resonators, and photonic crystals. Here we theoretically demonstrate that nonlinear self-organization can be exploited to freeze optical beams in bulk near-zero-index media, thus enabling three-dimensional self-trapping of still light without the need of optical resonators. Light is stopped to a standstill owing to the divergent wavelength and the vanishing group velocity, effectively rendering, through nonlinearity, a positive-epsilon trapping cavity carved in an otherwise slightly-negative-epsilon medium. By numerically solving Maxwell’s equations, we find a soliton-like family of still azimuthal doughnuts, which we further study through an adiabatic perturbative theory that describes soliton evaporation in lossy media or condensation in actively pumped materials. Our results suggest applications in optical data processing and storage, quantum optical memories, and soliton-based lasers without cavities. Additionally, near-zero-index conditions can also be found in the interplanetary medium and in the atmosphere, where we provide a complementary explanation to the rare phenomenon of ball-lightning.

Spatial and temporal self-trapping^1^,^2^,^3^,^4^ occur in several optical systems, including photorefractive media^5^,^6^, liquid crystals^7^,^8^, and metamaterials^9^,^10^. Remarkably, nonlinearity can act simultaneously on temporal and spatial domains to compensate for both diffraction and dispersion, thus enabling the formation of light bullets, spatio-temporal doughnuts, and X-shaped waves^11^,^12^,^13^,^14^,^15^.

Physical systems enabling either slow or fast light^16^,^17^,^18^ naturally enhance radiation-matter interaction, thus boosting nonlinear processes that can be efficiently used for active light control^19^, all-optical switching, and modulation^20^,^21^. In particular, near-zero-index (NZI) media can slow down light propagation^22^,^23^,^24^ and enable extreme nonlinear dynamics^25^, enhanced second and third harmonic generation^26^, active control of tunneling^27^, optical switching, and bistable response^28^. These materials naturally exist in nature, for example plasmas, transparent conductors, and metals near their bulk plasma frequency 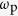
29. Besides, they can be artificially realized as waveguides close to modal cutoff^30^, using surface phonon polaritons in GaAs quantum wells^31^, or by engineering subwavelength metallic nanowires, nano-spheres, or nano-circuits embedded in dielectric matrices. The latter strategy has enabled the development of epsilon-near-zero (ENZ) metamaterials, which have been investigated for applications such as enhanced transmission^32^, cloaking^33^, energy squeezing in narrow channels^34^, and subwavelength imaging^35^,^36^. The ENZ regime is inevitably associated with high dispersion and is therefore accompanied by absorption, which can be suppressed by embedding externally pumped active inclusions in the NZI medium^37^.

Here we theoretically investigate self-organization of light in NZI media with Kerr-like instantaneous nonlinearity. In particular, we reveal the existence of fully confined doughnut-shaped solitons with vanishing Poynting vector and angular momentum. In practice nonlinearity enables digging a three-dimensional cavity for light, which in turn remains frozen and self-trapped. We study the effect of loss on stationary light doughnuts by developing a fully numerical soliton perturbative theory, finding that they evaporate over time due to absorption: their amplitude decreases, their frequency blueshifts slightly, and their radius increases. Conversely, if externally pumped active inclusions with inversion of population are embedded within the NZI medium, the opposite scenario takes place and azimuthal doughnuts condensate over time. These findings demonstrate the possibility to freeze light beams in ENZ media, with potential applications in optical data processing and storage, quantum optical memories, and NZI lasers operating without cavities. Interestingly, ENZ conditions are found also in the interplanetary medium and in the atmosphere, and we argue that our theoretical results may provide insight into ball-lightning (BL) formation^38^,^39^,^40^.

## Results and Discussion

### Still light

We consider a generic NZI medium with Drude temporal response and instantaneous Kerr-like nonlinearity (see Methods). Both of these ingredients ensue from free-particle temporal dynamics, which is characteristic of plasmas, metals, transparent conductors, and ENZ metamaterials, all examples of NZI media. In particular, Kerr-like nonlinearity naturally arises from the ponderomotive force in plasmas and metals^41^.

**Figure 1:**
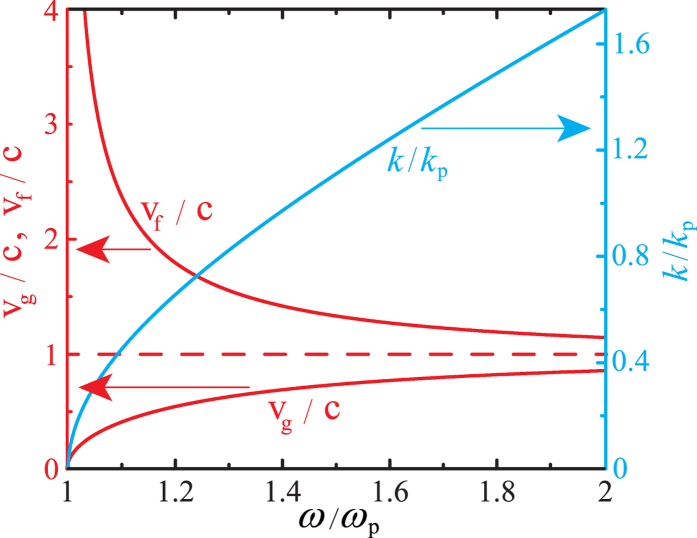
Dispersion relation *k*(*ω*) (cyan right *y*-axis) and phase and group velocities (v_*f*_ and v_*g*_, red left *y*-axis) of TEM waves in the linear loss-less limit. All quantities are plotted in dimensionless units: the angular frequency *ω* is normalized to the plasma frequency 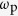, the wave-vector *k* is normalized to 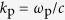, and the phase and group velocities are normalized to the speed of light in vacuum *c*. The red dashed line indicates the dispersion-less limit 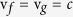.

In the linear limit, homogeneous transverse electromagnetic (TEM) waves are solutions of Maxwell’s equations with a complex electric field given by 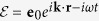, where 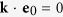. The angular frequency *ω* and the wave-vector **k** satisfy the dispersion relation 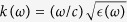, where *c* is the speed of light in free space and 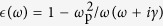 is the frequency-dependent dielectric constant, which is given by the Fourier transform of the Drude temporal response function 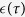 (see Methods). The material dispersion basically depends on two constants: the plasma frequency 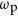 and the damping rate *γ*. The linear dispersion relation of TEM waves 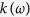 is depicted in Fig. 1 in the lossless limit 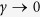, together with the phase and group velocities 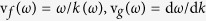. Note the cutoff of TEM waves at the plasma frequency 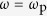, where the medium enters the ENZ regime, the phase velocity diverges, and the group velocity vanishes^22^,^23^,^24^.

### Homogeneous nonlinear modes

**Figure 2:**
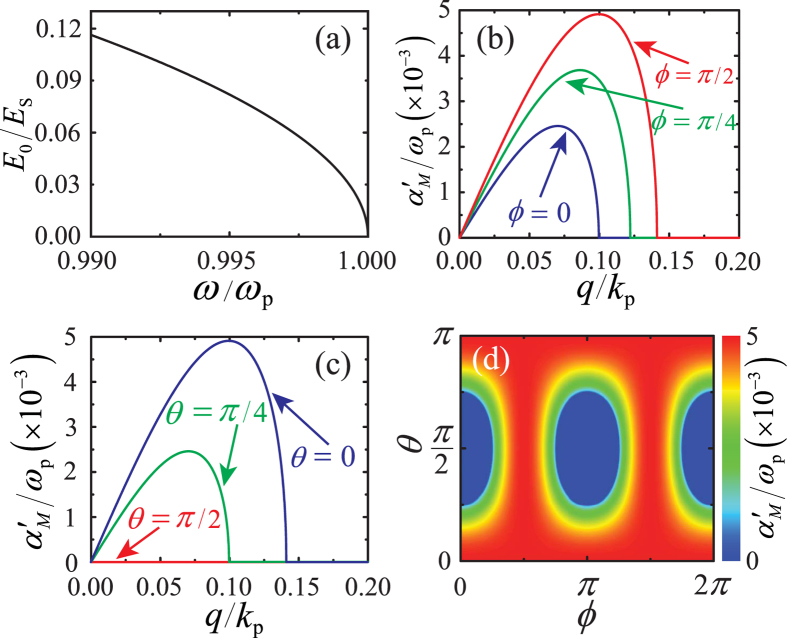
(**a**) Nonlinear dispersion of zero-index homogeneous modes existing for angular frequencies *ω* smaller than the cutoff 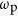. The electric field amplitude 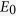 is normalized to the scaling electric field 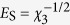. (**b**,**c**) Maximum instability growth 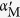 (normalized to the plasma frequency 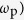 as a function of the perturbing wave-vector modulus *q* (normalized to 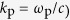 for several directions in the reciprocal space: (**b**) 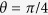, 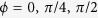, and **(c)** 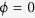, and 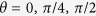. (**d**) Contour-plot of the maximum instability growth 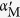 (normalized to the plasma frequency 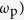 as a function of 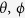 for a fixed perturbing wave-vector modulus 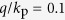.

Owing to the vanishing group velocity, nonlinear effects are dramatically enhanced in the ENZ regime^25^,^26^. For 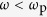, homogeneous modes with vanishing wave-number, infinite phase velocity, and zero group velocity can be found by neglecting damping and setting 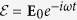, with 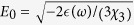, and 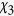 being the material’s Kerr coefficient (see Methods). The resulting dispersion relation is plotted in Fig. 2(a). We find zero-index homogeneous modes to have a cutoff at the plasma frequency 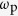, where the electric field amplitude drops to zero. In order to evaluate the stability of homogeneous modes, we perturb them with small-amplitude waves: 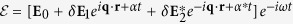, where 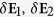 are the perturbation amplitudes with wave-vector **q** and temporal growth eigenvalue *α*. Inserting this expression in the Maxwell’s equations and retaining only the lowest-order terms in 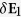 and 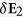, we find a homogeneous system of linear equations, whose non-trivial solutions are signaled by the vanishing of the secular determinant [see supplementary information (SI) for more details on the technical aspects of the theory]. This condition determines the complex temporal eigenvalues *α*. Instabilities are then associated with positive real parts of the eigenvalue *α*, indicating unbound amplification of the perturbation. We plot results of the stability analysis in Fig. 2(b–d), and in particular, we depict the maximum of the real part of the eigenvalue, 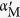. In analogy to standard modulation instability in 1D paraxial systems^3^, the gain spectrum of the perturbations is non-vanishing within a finite wave-vector window and is peaked at a characteristic wave-vector modulus. However, in contrast to 1D paraxial systems, the gain spectrum is 3D and has a non-trivial dependence on polar and azimuthal angles (*θ*,*ϕ*) of the perturbation wave-vector **q**.

### Still azimuthal doughnuts

**Figure 3:**
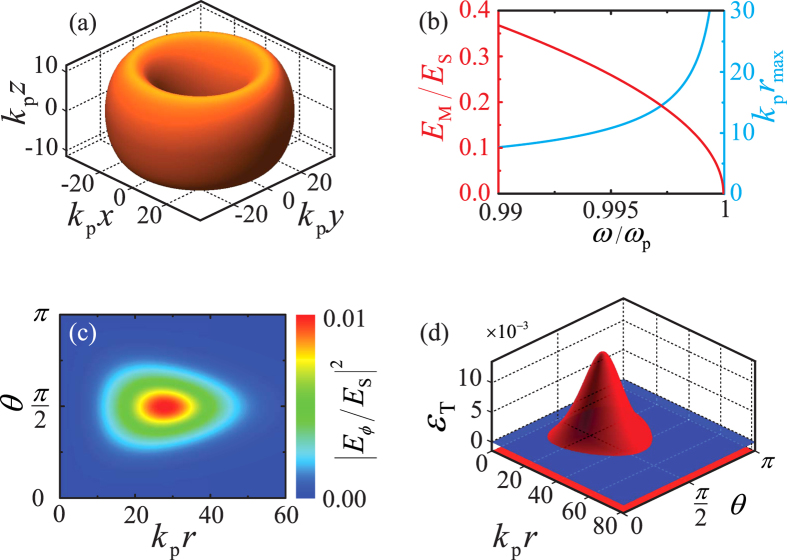
(**a**) Iso-surface 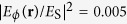 of a still doughnut with maximum squared amplitude 
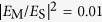
, where 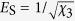 is the scaling field amplitude, excited at an angular frequency 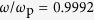. (**b**) Soliton maximum amplitude 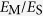 (red left *y*-axis) and its corresponding radius 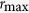 (cyan right *y*-axis) as a function of angular frequency 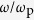. (**c**) Contour-plot in the *r*-*θ* plane of the dimensionless intensity profile 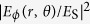 and (**d**) total dielectric permittivity profile 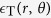 (red surface) associated with the still doughnut of (**a**). The blue plane in (**d**) represents the metal-dielectric transition plane 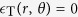. All quantities are plotted in dimensionless units: the angular frequency *ω* is normalized to the plasma frequency 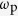, while spatial coordinates are normalized to the inverse of the plasma wave-vector 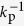.

The modulation instability scenario strongly suggests the presence of still 3D solitons in NZI media. In order to verify this hypothesis, we transform Maxwell’s equations into spherical coordinates and search for azimuthally-polarized solutions: 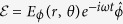. As Maxwell’s equations are invariant under a constant phase shift (see Methods), without any loss of generality we can assume that the electric field envelope is real 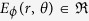, meaning that we are seeking non-propagating solutions which are not accompanied by a phase flow. Indeed, assuming that such solutions exist, we show that the Poynting vector vanishes thoroughly (see SI). Besides, we seek localized soliton-like solutions vanishing at 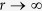 and at 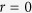, 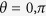 owing to the azimuthal polarization. Upon examination of the asymptotical expansion of Maxwell’s equations for 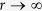, we find that 3D soliton-like azimuthal solutions can actually exist only in the ENZ regime (see SI). Thus, we discretize derivatives with respect to the radius 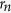 and the polar angle 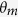 and then transform the differential wave equation for the electric field into a nonlinear algebraic system for the electric field amplitudes 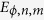 in the two-dimensional grid 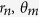 (see SI). We solve this nonlinear algebraic system by means of an iterative Newton-Raphson algorithm, and find a family of still azimuthal doughnuts [see Fig. 3(a)] for 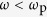, which presents a cutoff at 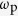, where the soliton loses localization and its amplitude vanishes. The frequency-dependent maximum amplitude and the corresponding radius of the still doughnut family are plotted in Fig. 3(b), while a *r*-*θ* contour-plot of the squared electric field profile 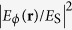 (normalized to the scaling field 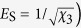 of the still doughnut at 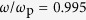 is depicted in Fig. 3(c). The total dielectric permittivity profile 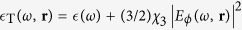 is shown in Fig. 3(d). Importantly, in the soliton existence domain 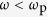, the linear dielectric constant is negative 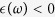, and thus, at long radius where the electric field amplitude is small, the NZI medium is metal-like. Conversely, in the volume around the radius 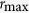 for which the electric field is maximum, nonlinearity is non-negligible and the total dielectric permittivity is positive 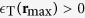 (dielectric-like). From here we see that the existence of still azimuthal doughnuts originates in the extraordinary ability of nonlinearity to dig a dielectric-like 3D cavity within a metal-like environment. This scenario is unique of NZI media, which prevent propagation of the fields outside the induced-dielectric trapping cavity. We emphasize that modulation instability enables the excitation of non-propagating solitons starting directly from unstable homogeneous waves with frequency falling in the ENZ regime.

### Doughnut evaporation/condensation

**Figure 4:**
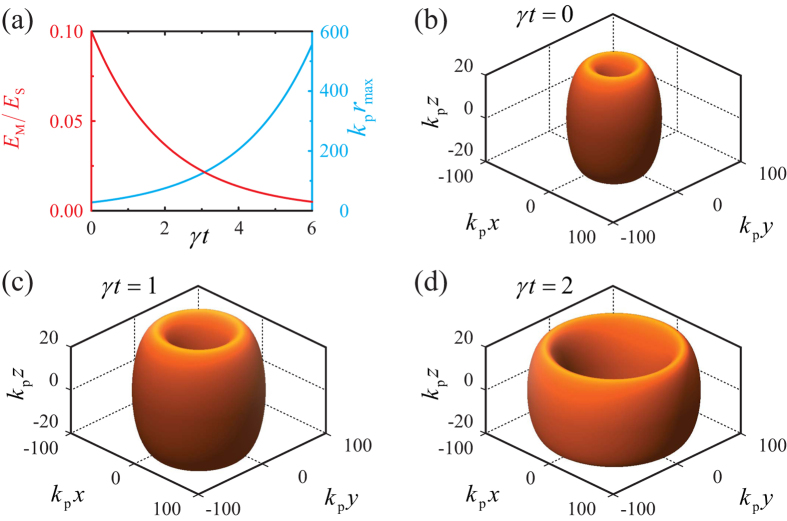
(**a**) Soliton maximum amplitude 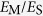 (red left *y*-axis) and its corresponding dimensionless radius 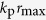 (cyan right *y*-axis) as a function of dimensionless time *γt*, where *γ* is a phenomenological damping. (**b–d**) Iso-surfaces 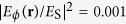 of the time-evolving doughnut with initial condition as in Fig. 3(a) for (**b**) 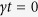, (**c**) 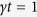, and (**d**) 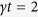.

In standard transparent media, the main quantity accounting for optical propagation is the Poynting vector, representing the temporal rate of energy transfer per unit area. For our trapped solitons, the Poynting vector is thoroughly vanishing (see SI), so we describe doughnut self-trapping through the optical-cycle-averaged density of electromagnetic energy. Now, if absorption is taken into account, the energy density is expected to be damped and vanish exponentially over time. A numerical verification of this hypothesis could consist in temporally evolving Maxwell’s equation with the doughnut initial condition. However, temporal evolution requires nonlinear 3D finite-difference-time-domain (FDTD) numerical simulations, which are computationally demanding. Besides, traditional approaches used in dielectric and plasmonic waveguides^42^,^43^,^44^ relying on the slowly-varying-envelope approximation (SVEA) can not be used, as the SVEA does not hold in the ENZ regime^25^. Instead, we have developed a soliton perturbation theory (see SI) capable of accounting for both damping and amplification (e.g., in systems containing externally pumped active inclusions within the NZI medium) under the assumption that (i) damping 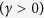 or (ii) gain 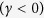 are much smaller than the soliton angular frequency *ω*. We further assume that the temporal evolution of the still doughnut adiabatically follows the soliton family, finding that the soliton amplitude (i) decays or (ii) increases over time following the exponential law 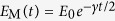, where 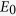 is the initial field amplitude and *γ* is a phenomenological absorption/pumping rate. Accordingly, the doughnut (i) expands and blueshifts or (ii) shrinks and redshifts in either case (see SI). The time-dependent field amplitude (blue left *y*-axis) and doughnut radius (red right *y*-axis) are plotted in Fig. 4(a) for a representative example, along with three snap-shots of the iso-surface 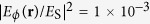 at different times in Fig. 4(b–d), where we have assumed as initial condition the doughnut of Fig. 3(a,i) damping 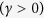 (For the full temporal evolution see movie in the SI). The doughnut evaporates over time, as its amplitude decreases and its radius increases. The gain scenario (ii) 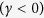 can be interpreted by inverting the temporal direction, so that the doughnut condensates over time, as its amplitude increases and its radius decreases.

### Ball-lightnings?

BLs are rare lightning events with hitherto unknown theoretical explanation^38^,^39^,^40^. BLs emit broadband radiation and can either propagate or stand still. Initially considered as myth, BLs have puzzled scientists for centuries and their existence has been questioned until the first recent experiment able to measure their spectrum^40^. Understanding of the nature of BLs is still unsatisfactory as they can not be easily reproduced in laboratory. Among the several theories trying to explain their nature, the so-called maser-caviton theory^38^ suggests that BLs are localized high-field solitons forming a cavity surrounded by plasma. Indeed, during thunderstorms, atmosphere can get ionized and become a NZI medium with a plasma frequency falling in the terahertz-microwave spectral region, where rotational levels of water can be excited. The ensuing emitted radiation is thought to remain self-trapped and heat up the air, thus emitting broadband blackbody radiation^38^. This theory explains several aspects of BLs, e.g., their typical size and their motion due to plasma density perturbations, but it does not provide any quantitative description of the self-induced soliton cavity. Following our rigorous calculations, as suggested by the maser-caviton theory, we speculate that BLs may actually ensue from a self-organization process in the ENZ regime, where we theoretically demonstrate the existence of still doughnut solitons, as discussed above. The actual spherical shape of BLs observed in experiments^40^ may be due to mixed polarization, heating and higher order nonlinear effects, or the intrinsically incoherent nature of radiation emitted in the atmosphere. The ENZ condition would explain the infrequency of the phenomenon and provides an insightful signature for experimental investigations.

## Conclusions

Our investigation of self-organization phenomena in NZI media with cubic nonlinearity has resulted in the demonstration that zero-index nonlinear waves are unstable in all spatial directions and that still azimuthally polarized self-trapped doughnuts can be excited. We have discussed the existence domain of this 3D soliton family with thoroughly vanishing Poynting vector and provided details on its characteristics. Besides, we have studied the effect of loss/amplification, finding that still light doughnuts evaporate/condensate over time, respectively. Our model applies to any NZI medium with cubic nonlinearity and our results are universal as they are rescaled to the relevant physical quantities (plasma frequency 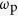, plasma wave-vector 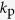, Kerr coefficient 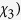 of any specific medium in this regime (e.g., metals, transparent conductors, plasmas, and metal-dielectric ENZ metamaterials). Our findings pave the way for the development of novel applications in optical data processing and storage, the realization of quantum optical memories, and the design of soliton-based lasers without cavities. Incidentally, NZI conditions can be found also in the interplanetary medium and in the atmosphere, and we have discussed possible relationships between our results and ball-lightning formation.

## Methods

### Model

In our investigations we have considered a generic NZI medium with Drude temporal response and instantaneous Kerr-like nonlinearity. Both of these ingredients ensue from free-particle temporal dynamics, which is characteristic of plasmas, metals, transparent conductors, and ENZ metamaterials, all examples of NZI media. In particular, Kerr-like nonlinearity naturally arises from the ponderomotive force in plasmas and metals^41^, and is well represented by the constitutive relation between the displacement vector 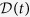 and the electric field 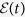:


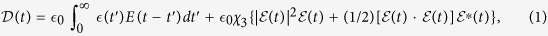


where 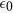 is the vacuum permittivity, 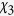 is the nonlinear susceptibility of the medium, 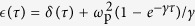 is the Drude temporal response function, 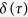 is the Dirac delta-function, 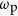 is the plasma frequency, and *γ* is the temporal damping rate due to inelastic collisions. Optical propagation is governed by the wave equation


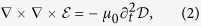


where 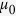 is the vacuum permeability.

### Still homogeneous waves

Homogeneous nonlinear modes with vanishing group velocity and diverging phase velocity have been calculated by inserting the Ansatz 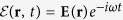 in Eq. (2), which in turn enables the calculation of the nonlinear dispersion.

In order to evaluate the stability of homogeneous modes, we have perturbed them with small-amplitude waves


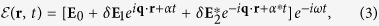


where 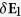 and 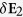 are the perturbation amplitudes with wave-vector **q** and temporal growth eigenvalue *α*. We have thus numerically calculated the complex eigenvalues *α* (which real parts represent the instability growth rates) of the ensuing homogeneous system of algebraic equations (see SI).

### Still azimuthal doughnuts

Given the isotropic nature of the system, the most natural coordinates to calculate 3D solitons are spherical 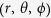, where *r* is the modulus of the position vector, and 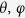 are its polar and azimuthal angles (see SI). In our calculations we have assumed that the electric field does not depend on the azimuthal angle, and thus it is polarized along the azimuthal direction 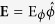. Still non-paraxial soliton-like solutions of Maxwell’s equations have been calculated numerically by transforming the continuous variables 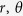 into a discrete two-dimensional grid 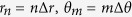 with steps 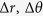, and the azimuthal electric field 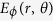 into an ordered vector 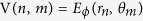. Approximating derivatives by finite differences, Eq. (2) becomes a nonlinear system of algebraic equations, which we have numerically solved through the Newton-Raphson method.

### Doughnut evaporation/condensation

We have accounted for the effect of damping/amplification in the temporal domain through an electric field amplitude 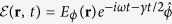 oscillating with angular frequency *ω* and exponentially decaying/increasing over time at a rate 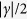. The soliton perturbative theory is then developed by assuming that, under the assumption of small damping/amplification 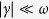, at every time *t*, the field pattern adiabatically follows the unperturbed soliton family with time-dependent maximum amplitude 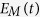, radius 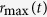, and angular frequency 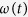. Inserting the expression for the electric field 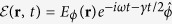 into Eqs. (1) and (2), and making use of the adiabatic approximation, one obtains the time-dependent soliton parameters 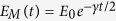, and


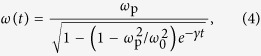



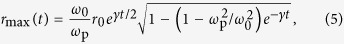


where 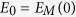, 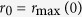, and 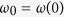 are the field amplitude, radius, and angular frequency of the soliton at the initial time 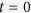, respectively.

## Additional Information

**How to cite this article**: Marini, A. and Abajo, F. J.G. Self-organization of frozen light in near-zero-index media with cubic nonlinearity. *Sci. Rep.* **6**, 20088; doi: 10.1038/srep20088 (2016).

## Supplementary Material

Supplementary Movie S1

Supplementary Information
