# Supplementary material for: Self-organization of frozen light in near-zero-index media with cubic nonlinearity
Source: Sci Rep. 2016 Feb 5;6:20088. doi: 10.1038/srep20088 (PMC4742830; doi:10.1038/srep20088)
Supplement: Supplementary Information [file srep20088-s2.pdf]

Self-organization of frozen light in near-zero-index media with cubic nonlinearity - SUPPLEMENTARY MATERIAL -

A. Marini[1,*] and F. J. García de Abajo[1,2]
[1]*ICFO-Institut de Ciencies Fotoniques, The Barcelona Institute of Science and Technology, 08860 Castelldefels (Barcelona), Spain and*
[2]*ICREA-Institució Catalana de Recerca i Estudis Avançats, Barcelona, Spain*
(Dated: December 16, 2015)

We provide additional information on zero-index homogeneous modes and their stability, as well as on azimuthal solitons of Maxwell's equations in spherical coordinates, for which we demonstrate that they have vanishing Poynting vector. Additionally, we give details on our theoretical derivations and computational methods, including a soliton perturbative theory used to account for loss/gain and explaining evaporation/condensation of azimuthal doughnuts.

I. GOVERNING LAWS

Before discussing specific aspects of self-organization, let us first examine the underpinning governing laws of light in near-zero-index (NZI) media with Drude temporal response and instantaneous Kerr-like nonlinearity. Optical propagation is determined by the double-curl Maxwell equations

$$\nabla \times \nabla \times \mathcal{E} = -\mu_0 \partial_t^2 \mathcal{D}, \tag{1}$$

where

$$\mathcal{D}(t) = \epsilon_0 \int_0^\infty \epsilon(t')\mathcal{E}(t-t')dt' + \epsilon_0\chi_3 \left\{ |\mathcal{E}(t)|^2 \mathcal{E}(t) + \frac{1}{2}\left[\mathcal{E}(t)\cdot\mathcal{E}(t)\right]\mathcal{E}^*(t) \right\} \tag{2}$$

is such that $\mathrm{Re}[\mathcal{D}(t)]$ is the displacement vector, μ_0 and ϵ_0 are the magnetic permeability and dielectric permittivity of vacuum, $\mathrm{Re}[\mathcal{E}(t)]$ is the electric field, χ_3 is the nonlinear susceptibility of the medium, $\epsilon(\tau) = \delta(\tau) + \omega_\mathrm{p}^2(1-e^{-\gamma\tau})/\gamma$ is the Drude temporal response function, $\delta(\tau)$ is the Dirac delta-function accounting for the instantaneous field, ω_p is the plasma frequency, and γ is a phenomenological damping rate. This temporal response function ensues straightforwardly from the classical equation of motion of a charged particle driven by an external electric field and damped through collisions with other particles [1].

II. HOMOGENEOUS NONLINEAR MODES

Monochromatic waves $\mathcal{E}(\mathbf{r},t) = \mathbf{E}(\mathbf{r})e^{-i\omega t}$, oscillating in time with angular frequency ω, satisfy the generalized Helmholtz equation

$$\nabla \times \nabla \times \mathbf{E} = \frac{\omega^2}{c^2}\left\{ \epsilon(\omega)\mathbf{E} + \chi_3\left[|\mathbf{E}|^2\mathbf{E} + \frac{1}{2}\left(\mathbf{E}\cdot\mathbf{E}\right)\mathbf{E}^* \right] \right\}, \tag{3}$$

where $c = 1/\sqrt{\epsilon_0\mu_0}$ is the speed of light in vacuum and $\epsilon(\omega) = 1 - \omega_\mathrm{p}^2/\omega(\omega+i\gamma)$ is the frequency-dependent dielectric constant, which is given by the Fourier transform of the Drude temporal response function $\epsilon(\tau)$. Zero-index homogeneous modes can then be calculated by setting $\mathbf{E}(\mathbf{r}) = \mathbf{E}_0$ and by neglecting damping ($\gamma = 0$), thus achieving the nonlinear dispersion relation $E_0 = \sqrt{-2\epsilon(\omega)/(3\chi_3)}$, which is plotted in Fig. 2(a) of the main paper. Zero-index homogeneous modes exist only for $\omega < \omega_\mathrm{p}$, where $\epsilon(\omega) < 0$ and present a cutoff at the plasma frequency ω_p, for which the electric field amplitude goes to zero.

*Electronic address: andrea.marini@icfo.es

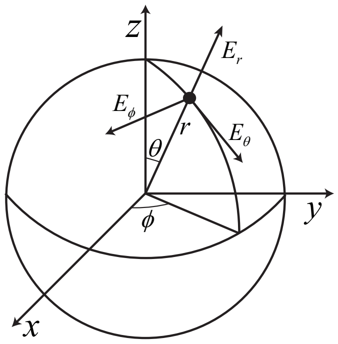

Figure S 1: Spherical coordinate system: radius r, polar and azimuthal angles θ and ϕ, and position vector $\mathbf{r}$. The electric field components E_r, E_θ, and E_ϕ along the curvilinear basis with unit vectors $\hat{r}$, $\hat{\theta}$, $\hat{\phi}$ are indicated.

Stability of homogeneous nonlinear modes

In order to evaluate the stability of homogeneous modes, we perturb them with small-amplitude waves

$$\mathcal{E}(\mathbf{r},t) = \left[\mathbf{E}_0 + \delta\mathbf{E}_1 e^{i\mathbf{q}\cdot\mathbf{r}+\alpha t} + \delta\mathbf{E}_2^* e^{-i\mathbf{q}\cdot\mathbf{r}+\alpha^* t}\right] e^{-i\omega t}, \tag{4}$$

where $\delta\mathbf{E}_1$ and $\delta\mathbf{E}_2$ are the perturbation amplitudes with wave-vector $\mathbf{q}$ and temporal growth eigenvalue α. Inserting Eq. (4) into Eqs. (1) and (2), and linearizing the ensuing system for the small perturbation amplitudes $\delta\mathbf{E}_1$ and $\delta\mathbf{E}_2$, we find

$$q^2\delta\mathbf{E}_1 - \mathbf{q}\left(\mathbf{q}\cdot\delta\mathbf{E}_1\right) + \frac{1}{c^2}(\alpha - i\omega)^2 \left\{\left[\epsilon(\omega+i\alpha) + \chi_3 E_0^2\right]\delta\mathbf{E}_1 + \chi_3\mathbf{E}_0\left[\mathbf{E}_0\cdot(2\delta\mathbf{E}_1+\delta\mathbf{E}_2)\right] + \frac{1}{2}\chi_3 E_0^2\delta\mathbf{E}_2\right\} = 0, \tag{5}$$

$$q^2\delta\mathbf{E}_2 - \mathbf{q}\left(\mathbf{q}\cdot\delta\mathbf{E}_2\right) + \frac{1}{c^2}(\alpha + i\omega)^2 \left\{\left[\epsilon(\omega-i\alpha) + \chi_3 E_0^2\right]\delta\mathbf{E}_2 + \chi_3\mathbf{E}_0\left[\mathbf{E}_0\cdot(\delta\mathbf{E}_1+2\delta\mathbf{E}_2)\right] + \frac{1}{2}\chi_3 E_0^2\delta\mathbf{E}_1\right\} = 0. \tag{6}$$

This homogeneous system of linear algebraic equations can be recast in matrix form as $\hat{\mathcal{M}}[\delta\mathbf{E}_1\ \delta\mathbf{E}_2]^T = 0$. Non-trivial solutions exist only if the determinant of the coefficient matrix is set to zero ($\det\hat{\mathcal{M}} = 0$), which yields a nonlinear equation for the complex temporal eigenvalues α of the linearized system. We numerically solve this equation through the Newton-Raphson method. Instabilities occur when the maximum real part of the eigenvalues α'_M is positive, as in this case perturbations are amplified. Results of our stability analysis are depicted in Figs. 2(b-d) of the main paper. We conclude that zero-index homogeneous waves are unstable along all directions and that the instability gain spectrum is peaked at a specific q.

III. AZIMUTHAL SOLITONS

Results of the stability analysis of homogeneous waves indicate the existence of a three-dimensional (3D) stationary mode with a particular spatial pattern that does not propagate and remains frozen in the medium. Note that, in the epsilon-near-zero (ENZ) regime ($|\epsilon(\omega)| << 1$), the double-curl operator $\nabla\times\nabla\times\mathbf{E}$ can not be approximated by $-\nabla^2\mathbf{E}$ because the divergence of the electric field $\nabla\cdot\mathbf{E}$ is non-negligible:

$$\nabla\cdot\mathbf{E} = -\frac{\chi_3}{\epsilon(\omega)}\nabla\cdot\left[|\mathbf{E}|^2\mathbf{E} + \frac{1}{2}\left(\mathbf{E}\cdot\mathbf{E}\right)\mathbf{E}^*\right]. \tag{7}$$

Indeed, while in standard photonic materials $\epsilon(\omega) \approx 1$, $|\chi_3 E^2| << 1$ and thus $|\nabla\nabla\cdot\mathbf{E}| << |\nabla^2\mathbf{E}|$, in NZI media this approximation generally does not hold owing to the small relative permittivity $|\epsilon(\omega)| << 1$. Consequently, we must retain the full-vectorial character of the double-curl equations, which anyway does not preclude the existence of non-paraxial solitons [2, 3]. Given the isotropic nature of the system, the most natural coordinates to calculate 3D solitons are spherical (r,θ,ϕ), where r is the modulus of the position vector, and θ,ϕ are its polar and azimuthal angles, see Fig. S1. The electric field is then written in the curvilinear basis $\mathbf{E} = E_r\hat{r} + E_\theta\hat{\theta} + E_\phi\hat{\phi}$, where $\hat{r},\hat{\theta},\hat{\phi}$ are the radial, polar and azimuthal unit vectors (see Fig. S1). In this coordinate system, the electric field can be

written as $\mathbf{E}(r,\theta,\phi)=\mathbf{e}(r,\theta)e^{im\phi}$, where m is the azimuthal charge accounting for orbital angular momentum. In our investigations, we have limited ourselves to $m=0$ case. Then, it can be directly verified from Maxwell's equations that the electric field is polarized along the azimuthal direction $\mathbf{E}=\mathrm{E}_\phi\hat{\phi}$ [4], and Eq. (3) reduces to

$$\partial_r^2\mathrm{E}_\phi+\frac{1}{r^2}\partial_\theta^2\mathrm{E}_\phi+\frac{2}{r}\partial_r\mathrm{E}_\phi+\frac{\cos\theta}{r^2\sin\theta}\partial_\theta\mathrm{E}_\phi-\frac{\mathrm{E}_\phi}{r^2\sin^2\theta}+\frac{\omega^2}{c^2}\left[\epsilon(\omega)+\frac{3}{2}\chi_3\left|\mathrm{E}_\phi\right|^2\right]\mathrm{E}_\phi=0. \quad (8)$$

Poynting vector

The time averaged Poynting vector is given by $\mathbf{S}=(1/2)\mathrm{Re}[\mathbf{E}\times\mathbf{H}^*]$, where the magnetic field can be expressed in terms of the curl of the electric field: $\mathbf{H}=\nabla\times\mathbf{E}/(i\mu_0\omega)$. Additionally, the time averaged Poynting vector of a purely azimuthal electric field is explicitly given by

$$\mathbf{S}=\frac{1}{2\mu_0\omega r}\left\{\mathrm{Re}\left[iE_\phi\partial_r\left(rE_\phi^*\right)\right]\hat{r}+\frac{1}{\sin\theta}\mathrm{Re}\left[iE_\phi\partial_\theta\left(\sin\theta E_\phi^*\right)\right]\hat{\theta}\right\}. \quad (9)$$

Note that Eq. (8) has purely real coefficients (as we are neglecting damping), and any of its solutions is characterized by a constant phase. Consequently, the Poynting vector vanishes everywhere, $\mathbf{S}=0$. The effect of loss/gain is treated in Sec. IV through a soliton perturbative theory.

Asymptotic behavior

Before accomplishing a the numerical study of soliton-like solutions of Eq. (8), it is useful to obtain further insight about their existence domain and their behavior in analytically treatable limits. In particular, in the limit $r\to 0$, nonlinear terms become negligible and Eq. (8) can be approximated by

$$\frac{1}{r^2}\partial_\theta^2\mathrm{E}_\phi+\frac{\cos\theta}{r^2\sin\theta}\partial_\theta\mathrm{E}_\phi-\frac{\mathrm{E}_\phi}{r^2\sin^2\theta}\approx 0. \quad (10)$$

Besides, as the polarization of the electric field is azimuthal, it is inherently required that $\mathrm{E}_\phi=0$ at the origin $r=0$ and along the z-axis ($\theta=0,\pi$), where the azimuthal polarization is not well defined. Moreover, we find that the electric field around the origin must behave like $E_\phi(r,\theta)\approx r^a\sin\theta$, where the exponent a remains unknown.

Conversely, in the limit $r\to\infty$, Eq. (8) can be approximated by

$$\partial_r^2\mathrm{E}_\phi+\frac{\omega^2}{c^2}\left[\epsilon(\omega)+\frac{3}{2}\chi_3\left|\mathrm{E}_\phi\right|^2\right]\mathrm{E}_\phi\approx 0, \quad (11)$$

so it admits exponentially decaying analytical solutions of the form $E_\phi\approx 4\sqrt{-\epsilon(\omega)/3\chi_3}\exp\left[-(\omega r/c)\sqrt{-\epsilon(\omega)}\right]$ only if $\epsilon(\omega)<0$ ($\omega<\omega_\mathrm{p}$), which coincides with the existence domain of homogeneous modes. Additionally, we find that localized solutions can exist only within a metal-like environment ($\epsilon(\omega)<0$). From this analysis it is clear that frozen (non-propagating) solitons can only exist in the ENZ regime, where focusing nonlinearity is sufficiently large to convert a metal-like domain into a dielectric-like one, thus digging a cavity for light that remains self-trapped.

Numerical calculation of frozen azimuthal solitons

Exact soliton-like solutions of Eq. (8) can be calculated numerically by introducing dimensionless radius $\tilde{r}=\omega r/c$ and electric field amplitude $\mathcal{A}=\sqrt{\chi_3}\mathrm{E}_\phi$. The continuous variables $\tilde{r},\theta$ are transformed into a discrete two-dimensional grid $\tilde{r}_n=n\Delta\tilde{r},\theta_m=m\Delta\theta$ with steps $\Delta\tilde{r},\Delta\theta$, while the amplitude $\mathcal{A}(\tilde{r},\theta)$ becomes an ordered vector $\mathrm{V}(n,m)=\mathcal{A}(r_n,\theta_m)$. The integer polar index m runs between in the interval $0<m<M=\pi/\Delta\theta$, while the radial index n runs in the $0<n<N$ range, where N needs to be large enough as to guarantee that the electric field vanishes with sufficient accuracy. Approximating derivatives by finite differences, Eq. (8) becomes the nonlinear algebraic system

$$\begin{aligned}&\frac{1}{\Delta\tilde{r}^2}[\mathcal{A}(n+1,m)-2\mathcal{A}(n,m)+\mathcal{A}(n-1,m)]+\frac{1}{\tilde{r}_n^2\Delta\theta^2}[\mathcal{A}(n,m+1)-2\mathcal{A}(n,m)+\mathcal{A}(n,m-1)]+\\&\frac{1}{\tilde{r}_n\Delta\tilde{r}}[\mathcal{A}(n+1,m)-\mathcal{A}(n-1,m)]+\frac{\cos\theta_m}{2\tilde{r}_n^2\sin\theta_m\Delta\theta}[\mathcal{A}(n,m+1)-\mathcal{A}(n,m-1)]-\frac{1}{\tilde{r}_n^2\sin^2\theta_m}\mathcal{A}(n,m)+\\&\left[\epsilon(\omega)+\frac{3}{2}\mathcal{A}^2(n,m)\right]\mathcal{A}(n,m)=0,\end{aligned} \quad (12)$$

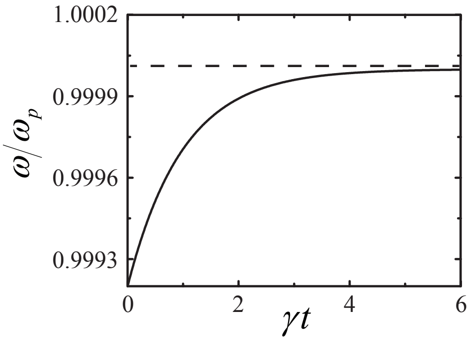

Figure S 2: Time-dependent soliton angular frequency (full line) and asymptotical limit $\omega = \omega_{\rm p}$ (dashed line). Parameters are identical to those of Fig. 4 of the main paper.

where $1 < n < N-1$ and $1 < m < M-1$ (i.e., these integers define the grid points in which Eq. (8) is evaluated). Thus, imposing the boundary conditions $\mathcal{A}(0,m) = \mathcal{A}(N,m) = \mathcal{A}(n,0) = \mathcal{A}(n,M) = 0$, we solve numerically the above nonlinear system using the Newton-Raphson iterative algorithm. The initial guess for the iterative method is set by following the hints of asymptotic expansions. We find convergence of doughnut-like solutions (with a tolerance on the norm of the nonlinear algebraic system down to 10^{-15}) with 10^4 points (100 points along θ and 100 points along r). Results of numerical calculations are reported in Fig. 3 of the main paper.

IV. SOLITON PERTURBATIVE THEORY

Homogeneous modes and still solitons are found in previous sections by neglecting damping. However, in real systems losses are present and need to be taken into account. As discussed in the main paper, they can be compensated for by embedding externally pumped active inclusions within NZI media [5]. Besides, if gain overcomes losses one gets lasing, which can be basically described in terms of a negative damping coefficient $\gamma < 0$. In order to describe the effect of (i) damping ($\gamma > 0$) or (ii) gain ($\gamma < 0$) on frozen self-trapped doughnuts, we here develop a soliton perturbative theory by assuming that dissipation/amplification happens at a much smaller temporal rate with respect to the soliton angular frequency $|\gamma| << \omega$. Note that this condition is thoroughly fulfilled by NZI media like plasmas, metals, transparent conductors, and ENZ metamaterials. Mathematically, the effect of loss/gain is accounted for by the imaginary part of the dielectric constant $\epsilon(\omega)$, which stems from the ansatz of CW monochromatic waves with purely real angular frequency ω. In this picture, the complex dielectric function $\epsilon(\omega)$ leads to a complex spatial phase pattern of the electric field. Here we use the alternative picture, where the effect of damping is considered in the temporal domain [6] through an electric field amplitude $\mathcal{E}(\mathbf{r},t) = E_\phi(\mathbf{r})e^{-i\omega t-\gamma t/2}\hat{\phi}$ oscillating with angular frequency ω and exponentially decaying/increasing over time at a rate $|\gamma|/2$. By inserting this expression in Eq. (2), one obtains

$$\mathcal{D}(\mathbf{r},t) = \epsilon_0 \left\{ \epsilon(\omega - i\gamma/2)E_\phi(\mathbf{r}) + \frac{3}{2}\chi_3 |E_\phi(\mathbf{r})|^2 E_\phi(\mathbf{r}) e^{-\gamma t} \right\} e^{-i\omega t-\gamma t/2}\hat{\phi}, \tag{13}$$

where $\epsilon(\omega - i\gamma/2) = 1 - \omega_{\rm p}^2/(\omega^2+\gamma^2/4)$ is purely real and can be approximated by $\epsilon(\omega - i\gamma/2) \approx \epsilon'(\omega) = 1-\omega_{\rm p}^2/\omega^2$ under the assumption $|\gamma| << \omega$. A soliton perturbative theory is then developed by assuming that, in the limit of small damping, at every time t, the field pattern adiabatically follows the unperturbed soliton family with time-dependent maximum amplitude $E_M(t)$, radius $r_{\rm max}(t)$, and angular frequency $\omega(t)$. Inserting the expression of the displacement vector in Eq. (1) and making use of the adiabatic approximation, one obtains the time-dependent soliton parameters $E_M(t) = E_0 e^{-\gamma t/2}$, and

$$\omega(t) = \frac{\omega_{\rm p}}{\sqrt{1-(1-\omega_{\rm p}^2/\omega_0^2)e^{-\gamma t}}}, \tag{14}$$

$$r_{\rm max}(t) = \frac{\omega_0}{\omega_{\rm p}} r_0 e^{\gamma t/2}\sqrt{1-(1-\omega_{\rm p}^2/\omega_0^2)e^{-\gamma t}}, \tag{15}$$

where $E_0 = E_M(0)$, $r_0 = r_{\rm max}(0)$, and $\omega_0 = \omega(0)$ are the field amplitude, radius, and angular frequency of the soliton at the initial time $t = 0$, respectively. Our main results from this soliton perturbative theory are depicted in

Fig. 4 of the main paper, which shows $E_M(t)$ and $r_{\max}(t)$, as well as the time-dependent intensity iso-surfaces. The soliton amplitude decays/increases exponentially, while its radius and angular frequency increase/decay with more involved temporal laws. The weak temporal dependence of the angular frequency is plotted in Fig. S2. Note that the weak frequency blueshift does not violate energy conservation, as the total energy decreases over time owing to the exponentially decaying electric field.

Video legend. The video in the supplementary information shows the temporal evaporation of a frozen soliton with initial squared amplitude $|E_{\rm M}/E_{\rm S}|^2 = 0.01$, where $E_{\rm S} = 1/\sqrt{\chi_3}$ is the scaling field amplitude, excited at an angular frequency $\omega/\omega_{\rm p} = 0.9992$. The orange doughnut-shaped surface indicates the iso-surface $|E_\phi(\mathbf{r})/E_{\rm S}|^2 = 0.001$.

[1] Ashcroft, N. W. & Mermin, N. D. Solid State Physics. (Saunders, Philadelphia, 1976).

[2] Ciattoni, A., Crosignani, B., Di Porto, P., and Yariv, A. Vectorial nonparaxial propagation equation in the presence of a tensorial refractive-index perturbation. *J. of the Opt. Soc. of Am. B* **22**, 1384-1394 (2005).

[3] Ciattoni, A., Crosignani, B., Di Porto, P., and Yariv, A. Azimuthally polarized spatial dark solitons: exact solutions of Maxwell's equations in a Kerr medium. *Phys. Rev. Lett.* **94**, 073902 (2005).

[4] Jackson, J. D. Classical Electrodynamics. (Wiley, New York, 1975).

[5] Rizza, C., Di Falco, A., and Ciattoni, A. Gain assisted nanocomposite multilayers with near zero permittivity modulus at visible frequencies. *Appl. Phys. Lett.* **99**, 221107 (2011).

[6] Archambault, A., Besbes, M., and Greffet, J.-J. Superlens in the time domain. *Phys. Rev. Lett.* **109**, 097405 (2012).
